# Prepectoral and subpectoral direct-to-implant breast reconstruction yield high cosmetic scores: Surgeon ratings favor prepectoral placement—secondary results from a randomized controlled trial

**DOI:** 10.1016/j.jpra.2025.10.008

**Published:** 2025-10-23

**Authors:** Frederik Gulmark Hansen, Diana Lydia Dyrberg, Camilla Bille, Jens Ahm Sørensen, Jørn Bo Thomsen

**Affiliations:** a Research Unit for Plastic Surgery, Odense University Hospital, Odense, Denmark; b Institute of Clinical Research, University of Southern Denmark, Odense, Denmark

**Keywords:** Direct-to-implant reconstruction, Breast reconstruction, Prepectoral implant placement, Subpectoral implant placement, Cosmetic outcomes, Breast animation deformity, Patient satisfaction, Surgeon evaluation, Randomized controlled trial

## Abstract

**Background:**

Direct-to-implant (DTI) breast reconstruction is increasingly used following mastectomy, with implants placed in a subpectoral or prepectoral plane. While subpectoral placement has been the conventional approach, prepectoral reconstruction is gaining popularity due to reduced risk of breast animation deformity (BAD) and potentially improved patient comfort. However, comparative data on cosmetic outcomes remain limited.

**Objectives:**

To compare patient- and surgeon-reported cosmetic outcomes following prepectoral and subpectoral DTI breast reconstruction as a secondary analysis of a randomized controlled trial.

**Methods:**

Forty-two patients undergoing post-mastectomy DTI breast reconstruction were randomized to receive either subpectoral or prepectoral implant placement. Cosmetic outcomes were evaluated using a 10-point numeric rating scale (NRS) by both patients and two consultants in plastic surgery at 3 and 12 months postoperatively. Data were analyzed using independent t-test.

**Results:**

A total of 60 breasts were reconstructed: 27 subpectoral and 33 prepectoral. At 3 months, patient NRS scores were 8.0 ± 1.9 (subpectoral) vs. 8.5 ± 1.9 (prepectoral) (*p* = 0.4).At 12 months, scores were 7.8 ± 1.7 and 8.4 ± 1.4, respectively (*p* = 0.2).Surgeon NRS scores were 8.2 ± 1.1 (subpectoral) vs. 8.8 ± 1.4 (prepectoral) at 3 months (*p* = 0.1), and 7.5 ± 1.2 vs. 8.7 ± 1.2 at 12 months (*p* = 0.0004).

**Conclusion:**

Cosmetic satisfaction was high in both groups at any given time point. While patient ratings were comparable, surgeons evaluated prepectoral implant placement superior at 12 months. These findings support prepectoral DTI reconstruction as a cosmetically favorable option in eligible patients.

## Introduction

Direct-to-implant (DTI) breast reconstruction has become an increasingly common method for breast reconstruction following mastectomy. It avoids the need for prolonged treatment by eliminating the waiting period associated with delayed reconstruction, and is further associated with improved body image, sexual well-being, and overall quality of life during the postoperative period.^1^^,^^2^ When performing DTI, the implant may be placed either beneath the pectoralis major muscle (subpectoral) or above it (prepectoral), with both methods having distinct benefits and drawbacks.

Subpectoral implant placement has traditionally been favored for a thicker implant coverage and thereby a lower risk of complications including implant visibility or rippling.^2^ However, positioning the implant beneath the pectoralis major muscle can result in breast animation deformity (BAD), as activation of the muscle exerts force on the implant through its attachment to the overlying capsule, leading to visible movement or distortion of the breast during muscle contraction.^3^^,^^4^ BAD is not only a cosmetic concern but can also result in discomfort, muscle twitching, and impaired shoulder mobility, particularly in physically active individuals.^5^^,^^6^

Prepectoral reconstruction has re-emerged as an attractive alternative, facilitated using acellular dermal matrices (ADMs) for implant support. By positioning the implant above the pectoralis major muscle, the pre-pectoral approach eliminates muscular distortion and preserves chest wall anatomy. Prepectoral implant placement has been shown to yield aesthetic outcomes comparable to subpectoral techniques, while offering additional benefits such as reduced postoperative pain and a lower risk of developing BAD.^3^^,^^7^, ^8^, ^9^

Given the potential functional and aesthetic implications of BAD, the choice of implant pocket in DTI reconstruction remains an important consideration. Ultimately, cosmetic outcome plays a central role in patient satisfaction and quality of life, making it a key endpoint in evaluating the success of DTI breast reconstruction.

In our previously published randomized controlled trial we demonstrated that prepectoral reconstruction substantially reduces the incidence and severity of BAD compared to subpectoral placement.^3^ As a secondary endpoint of that study, we collected patient- and surgeon-reported evaluations of the cosmetic result using a numeric rating scale (NRS). In the present manuscript, we present and analyze those cosmetic outcomes up to 12 months postoperatively, providing additional insight into how implant pocket choice may influence aesthetic satisfaction in DTI breast reconstruction.

## Methods

This randomized controlled trial RCT was conducted in accordance with the CONSORT guidelines^10^ and approved by the regional ethics committee (No. 24/22826). A total of 42 women undergoing mastectomy with immediate DTI breast reconstruction were enrolled and randomized 1:1 to receive either subpectoral or prepectoral implant placement. Randomization was stratified and performed using computer-generated block sequences. Detailed methodology, patient demographics, and primary outcomes are described in the original publication by Dyrberg et al.^3^

### Surgical technique

All patients received smooth, round silicone implants with the use of ADM for implant support. In the subpectoral group, the pectoralis major muscle was partially elevated by dividing its inferomedial insertion, creating a pocket for partial muscle coverage of the implant. The inferior pole of the implant was supported by a single sheet of ADM (10 × 16 cm) secured as a hammock from the muscle edge to the inframammary fold. In the prepectoral group, the implant was placed entirely anterior to the intact pectoralis major muscle. Two sheets of ADM were sutured circumferentially along the breast footprint, forming a total implant wrap and securing the prosthesis within a prepectoral pocket. Both techniques have been described in further detail in a previous publication. All procedures were performed by experienced consultant plastic surgeons following standardized institutional protocols.

### Cosmetic assessment

Cosmetic outcomes were assessed and photographically documented at 3 and 12 months postoperatively using a 10-point NRS, where one indicated the worst possible cosmetic result and 10 the best. Patients were asked to rate their satisfaction with the overall appearance of the reconstructed breast. If a patient had a bilateral reconstruction, they were asked to rate the two breasts independently. Two consultants in plastic surgery evaluated standardized photographic documentation of each breast reconstruction, assessing size, shape, projection, symmetry, and position of the breast mound relative to the chest wall.

### Statistical analysis

Descriptive statistics were used to summarize demographic and clinical data. Continuous variables, including NRS scores, were analyzed using independent t-tests. A *p*-value of <0.05 was considered statistically significant. All statistical analyses were performed using STATA version 16 (StataCorp, College Station, TX, USA).

## Results

A total of 60 breasts were reconstructed: 27 in the subpectoral group and 33 in the pre-pectoral group. At 3- and 12-month follow-up, 57 breast reconstructions (25 subpectoral and 32 prepectoral) were eligible for cosmetic assessment (supplemental figure 1). Two patients in the subpectoral group and one in the pre-pectoral group required implant removal due to complications unrelated to aesthetic concerns.

Patient demographics and baseline characteristics were comparable between groups as described in Dyrberg et al.^3^ In brief, the distribution of therapeutic versus prophylactic mastectomy was similar between groups. Mean age (50.0 ± 10.2 vs. 49.4 ± 10.9 years) and BMI (26.8 ± 2.25 vs. 25.5 ± 2.4 kg/m²) showed no significant differences. A greater proportion of patients in the subpectoral group underwent unilateral reconstruction (71.4 % vs. 42.9 %), though this difference was not statistically significant. The median duration of surgery, type of implant used, implant volume, and exposure to chemotherapy were also comparable across groups. All patients in the prepectoral group received silicone implants, while two patients in the subpectoral group received expanders. No statistically significant differences were observed in any demographic or treatment-related variable.

At 3 months postoperatively, patients rated the overall cosmetic outcome similarly in both groups, with a mean score of 8.0 ± 1.9 in the subpectoral group and 8.5 ± 1.9 in the prepectoral group (*p* = 0.4). These ratings remained comparable at 12 months, with scores of 7.8 ± 1.7 and 8.4 ± 1.4, respectively (*p* = 0.2). Surgeon assessments also demonstrated high overall cosmetic ratings. At 3 months, the mean scores were 8.2 ± 1.1 for subpectoral and 8.8 ± 1.4 for prepectoral reconstructions (*p* = 0.1). However, at 12 months, surgeon-assessed scores favored the prepectoral group significantly, with a mean of 8.7 ± 1.2 compared to 7.5 ± 1.2 in the subpectoral group (*p* = 0.0004) (Table 1).

**Table 1:** Patient- and surgeon-reported cosmetic scores.

	Patient	Surgeon	Patient	Surgeon
	3 months	3 months	12 months	12 months
Subpectoral (Mean ± SD)	8.0 ± 1.9	8.2 ± 1.1	7.8 ± 1.7	7.5 ± 1.2
Prepectoral (Mean ± SD)	8.5 ± 1.9	8.8 ± 1.5	8.4 ± 1.4	8.7 ± 1.2
Difference CI)	−0.4 (− 1.4 − 0.6)	−0.6 (−1.3 – 0.15)	−0.5 (−1.4 – 0.3)	−1.2 (−1.8 − (−0.5))
*p*-value	0.4	0.1	0.2	0.0004*

Cosmetic evaluation on a Numeric Rating Scale (NRS) from 1–10 by the patient herself and by the surgeon at 3- and 12-months follow-up.

⁎ Significant difference between sub-and prepectoral reconstructed group.

## Discussion

In this randomized controlled trial, both patients and surgeons rated the cosmetic outcomes of direct-to-implant breast reconstruction highly, regardless of whether the implant was placed in the subpectoral or prepectoral plane. Patient-reported satisfaction with the aesthetic result remained consistently high at both 3 and 12 months postoperatively, with no statistically significant differences between groups. However, surgeon assessments revealed a different trend. While both techniques were rated positively, prepectoral reconstructions were rated significantly higher at 12 months. Although both values fall within a range considered aesthetically acceptable on the 10-point NRS, the difference indicates that surgeons perceived a superior cosmetic result with prepectoral implant placement at longer follow-up. These assessments likely reflect greater appreciation of subtle differences in symmetry, contour, and projection. The observed difference in surgeon ratings may, however, reflect the current trend toward prepectoral reconstruction, which could introduce some degree of evaluator bias. Surgeons, particularly in academic and reconstructive settings, may be more inclined to favor prepectoral results due to increasing familiarity and enthusiasm for this approach. Although standardized photographic assessments were used to reduce subjectivity, and raters were not involved in the original procedures, it remains possible that prevailing preferences subconsciously influenced evaluations. This potential bias should be considered when interpreting surgeon-reported outcomes.

The cosmetic outcome remains a central element of patient satisfaction in breast reconstruction, and our findings are in line with previous studies indicating high satisfaction in both subpectoral and prepectoral approaches.^9^ In the present trial, patient-reported cosmetic scores were consistently high in both groups at 3 and 12 months, with no statistically significant differences. These results are supported by a recent meta-analysis including six studies, where BREAST-Q scores on the “satisfaction with breast” subscale were favorable in both techniques—77.3 % in the prepectoral group and 71.1 % in the subpectoral group—though the difference was not statistically significant.^9^ Interestingly, two of the included studies that examined aesthetic satisfaction more specifically reported significantly better outcomes in the prepectoral group. While pooled analysis did not confirm this difference across all studies, considerable heterogeneity suggests variation in patient populations, surgical techniques, or timing of the evaluation, which may influence the aesthetic rating. Our findings similarly point to a potential aesthetic advantage of prepectoral placement as observed in surgeon ratings at 12 months, despite patients themselves reporting equal satisfaction. Together, these results highlight that both techniques can achieve good cosmetic results, but subtle differences may be more detectable to trained observers or emerge more clearly in specific patient subgroups.

These findings suggest that, from the patient perspective, the choice of implant pocket may not substantially influence overall satisfaction with breast appearance.

This analysis represents a secondary outcome from a previously published randomized trial, in which the primary focus was BAD. That study demonstrated a clear advantage of prepectoral reconstruction in reducing the incidence and severity of BAD.^3^ Patient demographics, surgical technique, and perioperative care were standardized between groups, and the cosmetic evaluation was performed in a blinded, systematic fashion using validated measures. While the randomized controlled design strengthens the validity of these findings, it is important to interpret secondary outcomes with some caution. The sample size of this trial was originally calculated to detect differences in the primary outcome (BAD). An interim analysis was performed after 42 patients had been enrolled, based on compelling data from retrospective studies and systematic reviews. Due to the highly significant difference in BAD between groups observed at that point, enrollment was closed early. As a result, this secondary cosmetic analysis may be underpowered to detect subtle differences in patient-reported outcomes, and the results should be interpreted accordingly. Another limitation is that while prepectoral reconstruction was always performed as direct-to-implant, a subset of patients in the subpectoral group initially required a tissue expander before definitive implant placement. This introduces a degree of heterogeneity that may have influenced cosmetic ratings, although all final evaluations were performed after exchange to the permanent implant.

Finally, cosmetic outcomes were assessed using a simple numeric rating scale. While practical and reproducible, this approach lacks the psychometric validation of established tools such as the BREAST-Q Animation Deformity Module.^11^ Importantly, at the time our study was designed, the specific BREAST-Q module for animation deformity and related aesthetic outcomes had not yet been developed nor translated to Danish. Future studies would benefit from incorporating such validated patient-reported outcome measures to capture a more comprehensive view of aesthetic satisfaction.

Nonetheless, the randomized setting, prospective design, and consistent follow-up provide valuable insight into the aesthetic impact of implant pocket choice in DTI breast reconstruction.

Taken together, these findings suggest that prepectoral reconstruction offers at least equivalent—and potentially superior—aesthetic results compared to subpectoral placement, particularly from the perspective of expert observers. However, the high level of patient satisfaction observed in both groups underscore that either approach can produce acceptable cosmetic outcomes when performed in appropriately selected patients. If the patient is a candidate for DTI breast reconstruction. The choice between prepectoral and subpectoral primarily depends on the thickness of the skin flaps. This can be evaluated preoperatively by palpation using a pinch-test, which should be at least 1.5 cm and preferably 2 cm. However, a preoperative MRI adds additional information visualizing the thickness of the subcutaneous tissue between the skin and the glandular tissue an also gives the surgeon a roadmap for the dissection plane between the glandula and the subcutaneous tissue.

The need for fat grafting in secondary procedures following prepectoral direct to implant breast reconstructions is correlated to the thickness, quality and viability of the mastectomy flaps. Thinner flaps with poor perfusion are associated with a larger risk for additional fat grafting procedures to correct the aesthetic outcome and especially rippling. If the flaps are too thin, severe rippling is unavoidable. If the flaps are between 1.5 and 2 cm, the patient should be informed that fat grafting is probably needed in secondary procedures to avoid rippling over time. When 2 cm or more, fat grafting is rarely indicated.

Future studies with larger cohorts and longer follow-up are needed to confirm whether the cosmetic benefits observed by surgeons in the prepectoral group persist over time and translate into measurable improvements in long-term patient satisfaction.

## Conclusion

In this secondary analysis of a randomized trial, cosmetic outcomes were rated highly by both patients and surgeons in both the subpectoral and prepectoral groups at 3 and 12 months postoperatively. While patient satisfaction remained consistently high regardless of implant placement, surgeon-rated aesthetic scores were significantly higher for prepectoral reconstructions at 12 months. These findings suggest that both approaches can achieve satisfactory cosmetic results, but that prepectoral DTI reconstruction may offer a modest aesthetic advantage as perceived by clinicians. These findings support prepectoral DTI reconstruction as a cosmetically favorable option when feasible. However, as this study was underpowered to detect small differences in patient-reported satisfaction and because cosmetic evaluation is inherently subjective, these results should be interpreted with caution.

## Ethics statement

The study was approved by the Regional Committees on Health Research Ethics for Southern Denmark (approval no.: S-20160160) and performed in accordance with the principles of the Declaration of Helsinki. Written informed consent was obtained from the included participants. The study has been prospectively registered at clinicaltrials.gov (identifier: NCT03143335) and the protocol has been published in trials (Direct-to-Implant Extracellular Matrix Hammock-based Breast Reconstruction; Prepectoral or Subpectoral?

## Declaration of AI

During the preparation of this manuscript, the authors used ChatGPT in order to improve readability and language. After using this tool, the authors reviewed and edited the content as needed takes full responsibility for the content of the publication.

## Declaration of competing interest

D.L.D. received a 1-year salary sponsor fee from Groupe Sebbin SAS. Apart from this, none of the authors have any disclosures in terms of financial and/or personal relationships with people or organizations that could influence this published work.

## Appendix Supplementary materials

Image, application 1
