# Supplementary material for: Prepectoral and subpectoral direct-to-implant breast reconstruction yield high cosmetic scores: Surgeon ratings favor prepectoral placement—secondary results from a randomized controlled trial
Source: JPRAS Open. 2025 Oct 23;48:12–7. doi: 10.1016/j.jpra.2025.10.008 (PMC12682047; doi:10.1016/j.jpra.2025.10.008)
Supplement: Supplementary file 1 [file mmc1.docx]

**Figure 1: CONSORT 2025 Flow Diagram**

Flow diagram of the progress through the phases of a randomised trial of two groups (that is, enrolment, intervention allocation, follow-up, and data analysis)

Lost to follow-up for primary outcome (n= 8):

Explantation due to infection (n= 2)

Insufficient mastectomy 🡪 Explantation (n= 1)

Did not want allocated treatment (n= 1)

Did not complete follow-up (n= 4)

Lost to follow-up for primary outcome (n= 3):

Insufficients mastectomy 🡪 explantation (n= 1)

Included by mistake (n= 19

Did not complete follow-up (n= 1)

Excluded (n= 16)

Not meeting inclusion criteria (n= 14)

Declined to participate (n= 2)

Analysis

Analysed for primary outcome (n= 21)

Randomised (n= 53)

Allocation

Follow-Up

Allocated to subpectoral implant placement (n= 24)

Allocated to prepectoral implant placement (n= 29)

Enrolment

Assessed for eligibility (n= 69)

Analysed for primary outcome (n= 21)
